# Metabolomic analysis in spondyloarthritis: A systematic review

**DOI:** 10.3389/fmicb.2022.965709

**Published:** 2022-09-02

**Authors:** Tianwen Huang, Yaoyu Pu, Xiangpeng Wang, Yanhong Li, Hang Yang, Yubin Luo, Yi Liu

**Affiliations:** ^1^Department of Rheumatology and Immunology, West China Hospital, Sichuan University, Chengdu, China; ^2^Rare Diseases Center, West China Hospital, Sichuan University, Chengdu, China; ^3^Institute of Immunology and Inflammation, Frontiers Science Center for Disease Related Molecular Network, West China Hospital, Chengdu, China

**Keywords:** spondyloarthritis, ankylosing spondylitis, metabolomics, biomarkers, dysbiosis

## Abstract

Spondyloarthritis (SpA) is a group of rheumatic diseases that cause joint inflammation. Accumulating studies have focused on the metabolomic profiling of SpA in recent years. We conducted a systematic review to provide a collective summary of previous findings on metabolomic profiling associated with SpA. We systematically searched PubMed, Medline, Embase and Web of Science for studies on comparisons of the metabolomic analysis of SpA patients and non-SpA controls. The Newcastle–Ottawa Scale (NOS) was used to assess the quality of the included articles. From 482 records identified, 31 studies were included in the analysis. A number of metabolites were differentially distributed between SpA and non-SpA cases. SpA patients showed higher levels of glucose, succinic acid, malic acid and lactate in carbohydrate metabolism, higher glycerol levels and lower fatty acid (especially unsaturated fatty acid) levels in lipid metabolism, and lower levels of tryptophan and glutamine in amino acid metabolism than healthy controls. Both conventional and biological therapy of SpA can insufficiently reverse the aberrant metabolism state toward that of the controls. However, the differences in the results of metabolic profiling between patients with SpA and other inflammatory diseases as well as among patients with several subtypes of SpA are inconsistent across studies. Studies on metabolomics have provided insights into etiological factors and biomarkers for SpA. Supplementation with the metabolites that exhibit decreased levels, such as short-chain fatty acids (SCFAs), has good treatment prospects for modulating immunity. Further studies are needed to elucidate the role of disordered metabolic molecules in the pathogenesis of SpA.

## Introduction

Spondyloarthritis (SpA) is a group of several related but phenotypically distinct disorders, including ankylosing spondylitis (AS), psoriatic arthritis (PsA), inflammatory bowel disease-associated SpA (IBD-SpA), reactive arthritis (ReA), juvenile idiopathic arthritis (JIA), enthesitis-related arthritis (ERA), and undifferentiated SpA (uSpA) (Taurog et al., 2016). According to the location of the joint involved, SpA can also be classified as either axial (axSpA) or peripheral (pSpA). This rheumatic disease mainly affects the back, pelvis, neck and some larger joints and manifests as pain, stiffness and fatigue. During the past decades, progress has been made in exploring the pathogenesis of SpA, genetic risk associations, HLA-B27-mediated pathology and the contribution of the type 3 immune response (Ranganathan et al., 2017). However, much remains to be fully elucidated. Moreover, although abundant evidence has proven that gut dysbiosis is common in SpA, especially AS (Yin et al., 2020; Zhou C. et al., 2020; Chen et al., 2021), the mechanisms mediating the crosstalk between the intestinal lumen and the immune system are still not completely defined.

Developments in molecular biology, along with the emergence of various new-omics techniques, have provided powerful tools for the advancement of etiological studies on SpA. Metabolomics is the large-scale study of small molecules, commonly known as metabolites, which directly reflect the underlying biochemical activity and state of cells/tissues. By the use of high-throughput techniques, metabolomics can be used not only to identify promising novel biomarkers but also to provide insights into etiology, leading to the development of therapeutic targets (Johnson et al., 2016; Wishart, 2016). In recent years, an increasing number of studies have been conducted to investigate the broad network of metabolites in SpA using various human biological samples, including serum, plasma, tissue, urine and feces (Gao et al., 2008; Fischer et al., 2012; Jiang et al., 2013; Zeft et al., 2014; Chen et al., 2015; Shao et al., 2016; Stoll et al., 2016; Butbul et al., 2020; Vernocchi et al., 2020; Funk and Becker, 2021). However, some studies have inconsistent results or even the opposite results. This may be caused by different standards for collecting samples and different measurement and analysis methods. Further analysis is needed.

Here, we conducted a systematic review of studies on metabolomics analysis of patients with SpA, aiming to summarize previous findings on the differences in the metabolic profiles between SpA and non-SpA participants, the dynamic alteration of metabolites in SpA before and after treatment and the differences among SpA subtypes. Furthermore, we discuss possible mechanisms by which altered metabolites are involved in the pathogenesis of SpA.

## Methods

This systematic review was registered with the International Prospective Register of Systematic Reviews (PROSPERO) database (registration no. CRD42022314657).

### Search strategy

This study was conducted following the recommendations of the Preferred Reporting Items for Systematic Reviews and Meta-Analyses Guidelines (PRISMA) (Moher et al., 2009). Published studies were retrieved after a literature search including the following 4 electronic databases: PubMed, Medline (ovidsp), Embase (ovidsp) and Web of Science. The literature search was performed in January 2022 (date of last search: January 3rd, 2022). The search strategy is shown in Supplementary Table 1. To ensure literature saturation, the reference lists of included studies or relevant reviews identified through the search were reviewed manually by 2 independent reviewers.

### Inclusion/exclusion criteria

The inclusion criteria were as follows: studies comparing the metabolomic profiling of human biological specimens from SpA patients to those of controls. We included SpA or any of the following subtypes: axSpA, pSpA, AS, PsA, IBD-SpA, JIA, ERA, ReA, and uSpA. No restrictions in terms of patient population age, disease stage or geographical location or publication dates of studies were applied. Whenever full-text articles were unavailable, abstracts were included if considered critically relevant. The exclusion criteria were as follows: (1) reviews, guidelines or editorials; (2) animal studies or *in vitro* studies; (3) studies using irrelevant omics techniques, such as transcriptomic and proteomics; and (4) studies in which cases only contained SpA patients without controls.

### Study selection and data collection process

Two reviewers independently screened the search results by title and abstract to determine which records were possibly eligible for inclusion in the systematic review. Subsequently, the full texts of all studies that potentially met the eligibility criteria were carefully assessed for final inclusion. Discrepancies in the final decision between reviewers were resolved by a reassessment performed by a third reviewer. The reasons for excluding studies were recorded. The data collected included the following: (1) publication information, including name of first author, year of publication and study geographic location; (2) patient demographics and characteristics close to the time of specimen collection, including age, sex, Bath Ankylosing Spondylitis Disease Activity Index (BASDAI), Ankylosing Spondylitis Disease Activity Score (ASDAS), C-reactive protein (CRP), erythrocyte sedimentation rate (ESR), and disease duration; (3) type of specimen; (4) methods used for metabolite identification and analysis; and (5) differentially distributed metabolites across comparison groups and alterations in major metabolic pathways associated with SpA.

### Quality assessment

The quality of the included studies was evaluated using the Newcastle–Ottawa Scale (NOS) (Wells et al., 2013), which is one of the most commonly used tools for quality assessments of non-randomized studies in systematic reviews. This scale consists of three domains, including the selection of the study groups, the comparability of the groups and the ascertainment of either the exposure or outcome of interest for case–control or cohort studies, respectively. Two well-trained authors applied NOS independently, and the discrepancies were resolved by discussion.

## Results

### Study selection and characteristics

**FIGURE 1:**
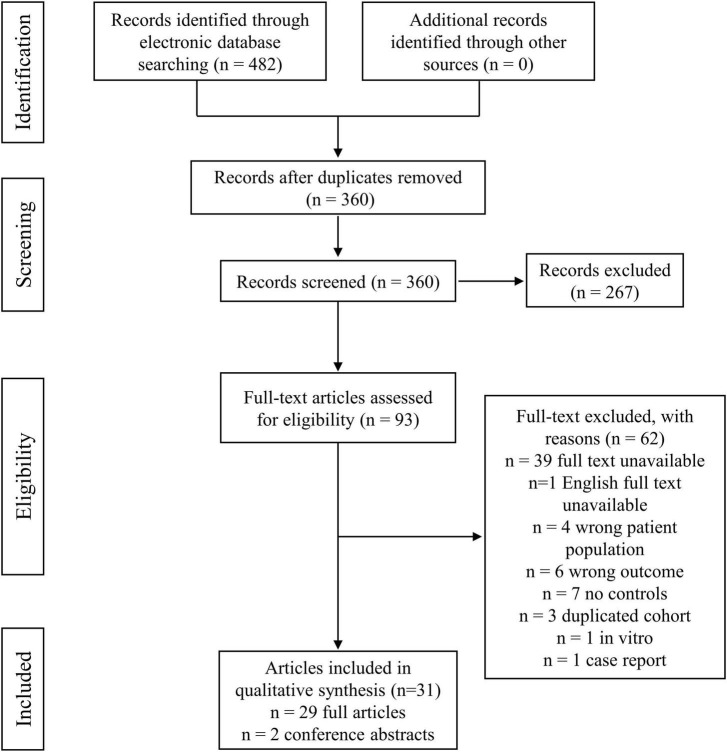
Flow diagram of study selection and data collection process.

The search results and the selection strategy are shown in Figure 1. Briefly, of the 482 records resulting from the initial electronic database search, 31 studies were finally included after rigorous screening according to the inclusion and exclusion criteria. The characteristics of the selected studies are summarized in Table 1. These studies included 1,176 SpA patients and 1,357 control participants. In the total 31 studies, 29 studies were original articles available as full text, while the other 2 studies (Zeft et al., 2014; Butbul et al., 2020) were conference abstracts. 22 of 31 studies conducted metabolomics comparisons between SpA cases and healthy controls. 12 of 31 studies compared SpA cases with cases of other rheumatic diseases, such as rheumatoid arthritis (RA), osteoarthritis (OA) and gout. 7 studies analyzed the dynamic alterations in metabolic profiles in SpA patients before and after treatment (Gao et al., 2008; Kapoor et al., 2013; Nanus et al., 2015; Butbul et al., 2020; Bogunia-Kubik et al., 2021; Funk and Becker, 2021; Gupta et al., 2021; Ou et al., 2021). Regarding the metabolomic analysis in different subtypes of SpA, 13 studies included AS patients (Gao et al., 2008; Fischer et al., 2012; Jiang et al., 2013; Chen et al., 2015; Shao et al., 2016; Wang et al., 2016; He et al., 2019; Zhou Y. et al., 2020; Bogunia-Kubik et al., 2021; Eryavuz Onmaz et al., 2021; Lv et al., 2021; Onmaz et al., 2021; Ou et al., 2021). 7 studies included PsA patients (Madsen et al., 2010; Kapoor et al., 2013; Armstrong et al., 2014; Nanus et al., 2015; Souto-Carneiro et al., 2020; Bogunia-Kubik et al., 2021; Rocha et al., 2021). 5 studies included JIA patients (Zeft et al., 2014; Stoll et al., 2016; Butbul et al., 2020; Vernocchi et al., 2020; Funk and Becker, 2021). 5 studies included ReA patients (Ahmed et al., 2019; Guleria et al., 2019; Li et al., 2019; Muhammed et al., 2020; Dubey et al., 2021). 2 studies included uSpA patients (Ahmed et al., 2019; Muhammed et al., 2020), 1 study included ERA patients (Stoll et al., 2016), 1 study included IBD-SpA (Berlinberg et al., 2021). 1 study enrolled cases with mixed subtypes of SpA without describing a specific subtype (Gupta et al., 2021). Only 3 studies compared different metabolic profiles among different subtypes of SpA (Ahmed et al., 2019; Bogunia-Kubik et al., 2021; Gupta et al., 2021).

**TABLE 1:** Characteristics of studies included in the systematic review.

Study	Region	Sample type	Analytical technique	SpA group							Control group		
				Subtype (No.)	Male/female	Age (yr)	Disease duration (yr)	CRP (mg/L)	ESR (mm/h)	BASDAI	Control (No.)	Male/female	Age (yr)
**SpA**													
Berlinberg et al. (2021)	United States	Colon biopsies	LC-MS	axSpA (21) CD-axSpA (12)	axSpA 11/10 CD-axSpA 5/7	axSpA 44.9 ± 12.1 CD-axSpA 51.4 ± 11.1	axSpA 9.9 ± 9.8 CD-axSpA 11.9 ± 8.0	NR	NR	axSpA 4.8 ± 2.4 CD-axSpA 5.1 ± 2.2	HC (24) CD (27)	HC 12/12 CD 14/13	HC 45.2 ± 11.8 CD 35.1 ± 18.1
Gupta et al. (2021)	India	Serum	^1^H NMR	SpA (81)	71/10	31.0 (23.0, 39.5)	6.0 (3.0, 12.0)	31.7 (4.3, 68.0)	60.0 (30.5, 92.5)	4.6 (2.6, 5.8)	HC (87)	72/14	32.2 ± 6.0
**AS**													
Gao et al. (2008)	China	Plasma	LC-MS and GC-MS	AS (15)	15/0	20.5 (7.0, 50.0)	At least 6 months	NR	NR	NR	HC (24)	Match with SpA groups	Match with SpA groups
Fischer et al. (2012)	United Kingdom	Serum	LC-MS	AS (18)	17/1	39.9 ± 12.8	NR	32.7 ± 37.3	42.0 ± 24.5	6.8 ± 1.9	HC (9)	8/1	40.9 ± 11.4
Jiang et al. (2013)	China	Serum	GC-TOF MS and UPLC-QTOF MS	AS (27)	27/0	31.0 (18.0, 55.0)	NR	13.9 ± 23.9	34.7 ± 26.2	NR	RA (27) OA (27) Gout (33) HC (60)	RA 0/27 OA 0/27 Gout 33/0 HC 30/30	RA 53.0 (40.0, 68.0) OA 58.0 (39.0, 73.0) Gout 51.0 (30.0, 69.0) HC 34.0 (25.0, 74.0)
Chen et al. (2015)	China	Serum	GC-MS	AS (33)	22/11	30.9 ± 7.8	At least 6 months	NR	NR	NR	HC (33)	19/14	33.9 ± 8.5
Shao et al. (2016)	China	Feces	^1^H NMR	AS (40)	24/16	34.0 ± 9.6	NR	7.6 ± 5.2	12.1 ± 8.7	NR	HC (34) RA (35)	HC 19/15 RA 10/25	HC 31.6 ± 10.2 RA 39.8 ± 7.9
Wang et al. (2016)	China	Plasma, Urine, ligament tissue	^1^H NMR	AS 44	38/6	31.8 ± 10.9	6.8 ± 3.5	NR	NR	3.2 ± 1.8	HC (44)	38/6	33.8 ± 9.7
He et al. (2019)	China	Feces	GC-MS	AS (49)	26/23	43.0 ± 9.6	NR	8.7 ± 5.2	13.6 ± 8.7	3.7 ± 2.1	HC (38)	20/18	43.1 ± 8.5
Zhou Y. et al. (2020)	China	Serum	UPLC-TQ-MS	AS (30)	20/10	34.0 (24.0, 46.0)	NR	19.9 ± 12.6	49.4 ± 23.1	NR	HC (30) RA (32)	HC 16/14 RA 14/18	HC 47.0 (21.0, 52.0) RA 44.0 (32.0, 60.0)
Bogunia-Kubik et al. (2021)	Poland	Serum	^1^H NMR	AS (29) PsA (23)	AS 22/7 PsA 14/9	AS 45.0 (26.0, 75.0) PsA 43.0 (29.0, 71.0)	AS 11.0 (1.0, 40.0) PsA 8.0 (2.0, 41.0)			AS 8.2 (5.7, 10.0) PsA 8.0 (6.0, 9.0)	RA (26)	5/21	55.0 (23.0, 74.0)
Eryavuz Onmaz et al. (2021)	Turkey	Serum	LC-MS	AS (85)	55/30	40.1 ± 9.4;	NR;	12.7 (3.0, 89.1);	20.7 (4.6, 103.5);	5.1 ± 1.1;	HC (50)	27/23	41.6 ± 6.8
Lv et al. (2021)	China	Saliva	GC-MS	AS 37	24/13	33.9 ± 1.9	14.5 (3.0, 59.8) months	7.1 (1.8, 15.8)	16.4 ± 2.7	3.1 (2.1, 3.9)	HC (41)	27/14	33.3 ± 1.7
Onmaz et al. (2021)	Turkey	Serum	LC-MS	AS 60	38/22	42.1 ± 8.1	9.7 ± 7.8	5.7 (1.4, 50.7)	14.5 (2.0, 60.0)	4.9 ± 1.7	HC (60)	35/25	42.9 ± 8.5
Ou et al. (2021)	China	Serum	LC-MS	AS 32	29/3	28.6 ± 7.5	94.3 ± 48.8 months	21.1 (12.4, 44.0)	29.5 (12.5, 46.5)	6.9 ± 2.0	HC (40)	37/3	27.1 ± 5.6
**PsA**													
Madsen et al. (2010)	Sweden	Plasma	LC-MS and GC-MS	PsA (20)	10/10	48.0 ± 12.0	15.0 ± 12.0	NR	NR	NR	RA (25)	9/16	51.1 ± 17.8
Kapoor et al. (2013)	United Kingdom	Urine	^1^H NMR	PsA (20)	10/10	NR	NR	NR	NR	NR	Baseline vs. 12 weeks after TNFi therapy		
Armstrong et al. (2014)	United States	Serum	GC-TOF MS	PsA (10)	5/5	15.0 ± 13.6	7.9 ± 6.8	NR	NR	NR	HC (10)	5/5	46.0 ± 15.0
Nanus et al. (2015)	United Kingdom	Urine	GC-MS	PsA (20)	10/10	48.0 ± 12.0	At least 6 months	14.2 ± 17.2	NR	NR	Baseline vs. 12 weeks after TNFi therapy		
Souto-Carneiro et al. (2020)	Germany	Serum	^1^H NMR	PsA (73)	44/29	56.2 (30.0, 78.0)	9.0 (0, 24)	6.7 ± 13.8	NR	NR	negRA (49)	10/39	64.2 (32.0, 83.0)
Rocha et al. (2021)	Spain	Synovium	MALDI-MSI	PsA 12	6/6	52.0 ± 13.0	NR	1.2 ± 1.8	NR	NR	OA (13)	2/11	73.0 ± 11.0
**JIA**													
Zeft et al. (2014)	United States	Exhaled breath	SIFT-MS	JIA (21)	NR	(5.0, 21.0)	NR	NR	NR	NR	HC (55)	NR	(5.0, 21.0)
Stoll et al. (2016)	United States	Feces	LC-MS	JIA/ERA (24)	12/12	14.0 (7.0, 17.0)	NR	NR	NR	NR	HC (19)	7/12	11.0 (7.0, 18.0)
Butbul et al. (2020)	Israel	Urine	GC-MS	JIA (11)	3/8	12.0 ± 6.2	6.3 ± 5.2	NR	NR	NR	HC (11)	Match with SpA groups	Match with SpA groups
Vernocchi et al. (2020)	Italy	Feces	GC-MS and ^1^H NMR	JIA (60)	16/44	7.0 ± 4.1	<6 months	1.4 ± 1.6	24.6 ± 20.4	NR	HC (25)	11/14	9.8 ± 2.9
Funk and Becker (2021)	United States	Plasma	GC-TOF-MS; Q-TOF-MS	JIA (30)	9/21	9.5 (5.0, 15.0)	NR	1.5 (0.5, 3.2)	16.0	NR	Baseline vs. 3 months after MTX therapy		
Ahmed et al. (2019)	India	Serum, synovial fluid	^1^H NMR	ReA (19) uSpA (13)	ReA 12/7	26.0	NR	NR	NR	NR	HC (18)	17/1	29.0
Guleria et al. (2019)	India	Serum	^1^H NMR	ReA (52)	44/8	29.0 ± 10.9	NR	7.4 ± 7.8	77.8 ± 37.6	NR	HC (82) RA (29)	HC 57/25 RA 2/27	HC 36.4 ± 9.3 RA 40.1 ± 11.8
Li et al. (2019)	United Kingdom	Serum, synovial fluid	LC-MS	ReA (7)	7/0	25.0 ± 4.3	9.0 ± 4.4 weeks	12.1 ± 15.9	9.8 ± 11.7	NR	HC (23) RA (20)	HC 9/14 RA 6/14	HC 48.0 ± 15.1 RA 54.1 ± 17.1
**ReA**													
Muhammed et al. (2020)	India	Serum, synovial fluid	^1^H NMR	ReA/uSpA (30) ReA (19) uSpA (11)	24/6	27.9 ± 9.1	NR	NR	NR	NR	RA (25) OA (21)	RA 5/20 OA 5/16	RA 41.5 ± 12.6 OA 59.8 ± 8.2
Dubey et al. (2021)	India	Synovial fluid	^1^H NMR	ReA (58)	49/9	29.1 ± 10.9	NR	NR	NR	NR	RA (21) OA (20)	RA 4/27 OA 4/16	RA 45.8 ± 12.6 OA 59.5 ± 8.3

This systematic review included 31 studies, in which a total of 1,176 SpA patients and 1,357 control participants were included. Among these studies, 13 studies included AS patients, 7 studies included PsA patients, 5 studies included JIA patients, 5 studies included ReA patients, 2 studies included uSpA patients, 1 study included ERA patients, 1 study included IBD-SpA and 1 study enrolled cases with mixed subtypes of SpA without describing a specific subtype. Descriptive statistics are presented as [mean ± SD or median (range)].

LC-MS, liquid chromatography–mass spectrometry; ^1^H NMR, proton nuclear magnetic resonance; GC-MS, gas chromatography–mass spectrometry; GC-TOF MS, gas chromatography time-of-flight mass spectrometry; UPLC-QTOF MS, ultra-high performance liquid chromatography-triple quadrupole mass spectrometry; UPLC-TQ-MS, ultra-performance liquid chromatography coupled with triple-quadrupole tandem mass spectrometry; MALDI-MSI, matrix-assisted laser desorption/ionization mass spectrometry imaging; SIFT-MS, selected ion flow tube mass spectrometry; Q-TOF-MS, quadrupole time-of-flight mass spectrometry; SpA, spondyloarthritis; axSpA, axial SpA; CD-axSpA, Crohn’s-axSpA; AS, ankylosing spondylitis; PsA, psoriatic arthritis; JIA, juvenile idiopathic arthritis; ReA, reactive arthritis; uSpA, undifferentiated SpA; HC, healthy control; RA, rheumatoid arthritis; negRA, seronegative RA; OA, osteoarthritis; CD, Crohn’s disease; yr, year; BASDAI, Bath Ankylosing Spondylitis Disease Activity Index; TNFi, TNF-alpha inhibitors; NR, not reported.

Biospecimens from different sources of tissues in SpA patients were applied for metabolic profiling and analysis. Nineteen studies assessed the metabolome by serum/plasma. Feces (Shao et al., 2016; Stoll et al., 2016; He et al., 2019; Vernocchi et al., 2020), urine (Kapoor et al., 2013; Nanus et al., 2015; Wang et al., 2016; Butbul et al., 2020), and synovial fluid (Ahmed et al., 2019; Li et al., 2019; Muhammed et al., 2020; Dubey et al., 2021) were commonly studied in these metabolomic researches. In addition, tissues such as ligament tissue (Wang et al., 2016), colon biopsies (Berlinberg et al., 2021), synovial membrane tissue (Rocha et al., 2021), saliva (Lv et al., 2021), and exhaled breath (Zeft et al., 2014) were also used for analysis in 1 study each. Depending on the characteristics of different tissues, metabolites were profiled by different platforms and methods. Most enrolled studies used proton nuclear magnetic resonance (^1^H NMR), liquid chromatography–mass spectrometry (LC–MS), and gas chromatography–mass spectrometry (GC–MS). Zeft et al. (2014) used selected ion flow tube mass spectrometry (SIFT-MS) for testing exhaled breath. Rocha et al. (2021) used matrix-assisted laser desorption ionization-mass spectrometry imaging (MALDI-MSI) for testing synovial membrane samples.

The results of the methodologic quality assessment by the NOS tool are summarized in Supplementary Table 2. The included studies had relatively good methodological quality. The definition and selection method for SpA cases were illustrated adequately in most studies. Nevertheless, control participants were explained only in one-third of cases. 18 of 31 studies showed fully comparable data between SpA patients and controls regarding the comparability of essential factors, such as sex and age. Data from 7 studies showed less comparison. Different distributions of both sex and age were observed in 6 studies, which might contribute to the risk of bias. The rate of unidentified metabolites for any reason in both groups was similar in all studies.

### Differences in metabolic profiling between spondyloarthritis patients and healthy controls

**FIGURE 2:**
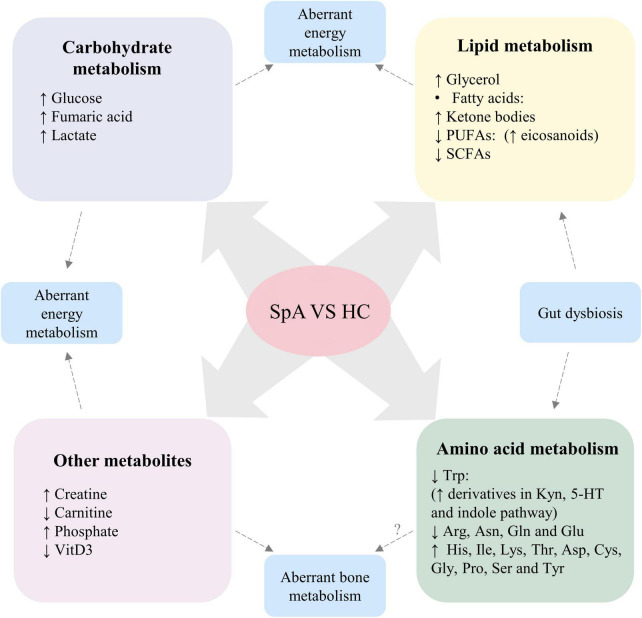
Altered metabolic profile consistently found in patients with SpA, compared with healthy participants. Dysregulation levels of carbohydrate and lipid metabolism, creatine and carnitine suggest aberrant energy metabolism in SpA patients. Altered levels of phosphate and VitD3 suggest aberrant bone metabolism in SpA patients, while the role of disturbed amino acid levels in the pathogenesis of aberrant bone metabolism requires further investigation. Moreover, gut dysbiosis of SpA contribute to altered levels of SCFAs, Trp and its derivatives. SpA, Spondyloarthritis; HC, healthy control; PUFAs, polyunsaturated fatty acids; SCFAs, short-chain fatty acids; VitD3, vitamin D3; Trp, tryptophan; Kyn, kynurenine; 5-HT, serotonin; Arg, arginine; Asn, asparagine; Gln, glutamine; Glu, glutamic acid; His, histidine; Ile, isoleucine; Lys, lysine; Thr, Threonine; Asp, aspartic acid; Cys, cysteine; Gly, glycine; Pro, proline; Ser, serine; Tyr, tyrosine.

Compared with healthy controls, distinct alterations of metabolic profiles, including metabolites involved in carbohydrate metabolism, lipid metabolism and amino acid metabolism, were found in patients with SpA (Figure 2).

#### Carbohydrate metabolism

Several studies consistently revealed that levels of metabolites involved in glycolysis and the tricarboxylic acid cycle (TCA cycle) were increased, such as glucose, glyceraldehyde, succinic acid, fumaric acid, and malic acid (Gao et al., 2008; Jiang et al., 2013; Shao et al., 2016; Ahmed et al., 2019; Guleria et al., 2019; Stahly et al., 2019; Lv et al., 2021; Onmaz et al., 2021). Two studies including patients with ReA showed decreased levels of pyruvate in serum (Ahmed et al., 2019; Guleria et al., 2019). Levels of lactate, an anaerobic oxidation product of glucose, were found to be elevated in the serum of SpA patients and positively correlated with disease activity (Gupta et al., 2021). In addition, upregulation of the levels of some products of carbohydrate metabolism, including mannose, glucuronic acid, gluconic acid, and propanedioic acid, were found in the serum and feces of SpA patients (Armstrong et al., 2014; Ahmed et al., 2019; He et al., 2019). Interestingly, Lewis et al. (2020) concluded that there was a positive correlation between pain and sugar intake in teens with active JIA, indicating the perturbation of carbohydrate metabolism in SpA.

#### Lipid metabolism

Lipid dysregulation was a common finding in most metabolomic studies of SpA, but these results were not consistent across studies. Two studies both reported elevated levels of glycerol in the serum of AS patients (Gao et al., 2008; Wang et al., 2016). Triglyceride levels were found to be increased in serum by Onmaz et al. (2021). Another analysis conducted by Wang et al. (2016) revealed downregulated levels in plasma and conversely upregulated levels in ligament tissue. One study conducted by Onmaz et al. (2021) showed elevated cholesterol levels in the serum of AS patients. He et al. (2019) showed lower levels of cholesterol in male AS patients than in healthy males by utilizing feces. Regarding lipoproteins, two studies independently indicated both decreased levels of low-density lipoprotein (LDL) and very low-density lipoprotein (VLDL) in serum (Guleria et al., 2019; Gupta et al., 2021). Onmaz et al. (2021) found upregulated levels of LDL and downregulated levels of high-density lipoprotein (HDL) in serum.

Fatty acids can be divided into saturated fatty acids and unsaturated fatty acids according to whether the hydrocarbon chain is saturated. Short-chain fatty acids (SCFAs) constitute an important part of saturated fatty acids because of their indispensable function in regulating the normal physiological functions of the body. Commonly altered SCFAs found in metabolomic studies include acetate, propionate, and butyrate. Two studies found increased acetate levels in the serum of SpA patients (Guleria et al., 2019; Gupta et al., 2021), while three studies found decreased acetate, propionate and butyrate levels in urine and feces (Shao et al., 2016; Wang et al., 2016; He et al., 2019). Moreover, levels of unsaturated fatty acids, especially polyunsaturated fatty acids (PUFAs), were consistently found to be decreased both in serum and colon tissue in four studies (Chen et al., 2015; Guleria et al., 2019; Berlinberg et al., 2021; Gupta et al., 2021). Ketone bodies, including acetoacetate, acetone and β-hydroxybutyrate, are intermediate metabolites of fatty acids during decomposition and oxidation in the liver. The levels of β-hydroxybutyrate and acetoacetate were consistently found to be increased in serum and saliva (Wang et al., 2016; Glaser et al., 2017; Ahmed et al., 2019; Guleria et al., 2019; Lv et al., 2021). However, the results of acetone were inconsistent in different studies. Studies including AS patients (Wang et al., 2016) and ReA patients (Ahmed et al., 2019) reported higher levels of acetone in patients compared with healthy control. Nevertheless, some other studies, including SpA patients (Gupta et al., 2021) and ReA patients (Guleria et al., 2019), reported lower levels of acetone than those in healthy controls.

Carnitine is an important molecule in lipid metabolism. Acetyl-L-carnitine, derived from carnitine, also plays an important role in fatty acid metabolism by facilitating the movement of acetyl-CoA into the matrices of mammalian mitochondria during the oxidation of fatty acids. Two studies including AS cases reported decreased levels of carnitine and acetyl-L-carnitine (Jiang et al., 2013; Zhou Y. et al., 2020), while one study found the converse results in JIA patients (Kiykim et al., 2015).

#### Amino acid metabolism

Perturbation of amino acid metabolism is also common in SpA and was reported in nearly half of the enrolled studies. Overall, the levels of ten amino acids, including histidine, isoleucine, lysine, threonine, aspartic acid, cysteine, glycine, proline, serine, and tyrosine, were found to be higher than those in healthy controls, regardless of sample type. Conversely, the levels of five amino acids: tryptophan, arginine, asparagine, glutamine, and glutamic acid, were found to be decreased. Inconsistent findings across studies were reported for other amino acids (Shao et al., 2016; Wang et al., 2016; Glaser et al., 2017; Zhou Y. et al., 2020; Gupta et al., 2021). Downregulation of phenylalanine (Shao et al., 2016; Zhou Y. et al., 2020), valine (Shao et al., 2016; Wang et al., 2016) and alanine (Wang et al., 2016; Glaser et al., 2017) levels were reported in one-third of the studies, respectively. Downregulation of leucine (Shao et al., 2016; Wang et al., 2016) and methionine (Shao et al., 2016; Gupta et al., 2021) levels were reported in two-fifths of the studies, respectively, while the other studies reported upregulation in the levels of these amino acids (Wang et al., 2016; Guleria et al., 2019; Zhou Y. et al., 2020; Gupta et al., 2021).

Several studies also reported altered levels of the primary derivatives of amino acids in SpA patients (Shao et al., 2016; Wang et al., 2016; Husni et al., 2017; Ahmed et al., 2019; Guleria et al., 2019; Zhou Y. et al., 2020; Onmaz et al., 2021). Both Husni et al. (2017) and Onmaz et al. (2021) found elevated levels of asymmetric dimethylarginine (ADMA), symmetric dimethylarginine (SDMA) and methylated arginine. Additionally, the altered levels of arginine and its derivatives in SpA patients remained after biological or conventional therapy when compared with the levels observed in healthy controls. TNF inhibitor treatment can reverse the aberrant metabolic state and promote the development of a metabolic state that is more similar to that of the controls more efficiently than that achieved with conventional therapy. Arginine, ornithine and citrulline are involved in the urea cycle. Consistent with the observed downregulation of arginine levels, several studies reported decreased levels of ornithine and citrulline in AS patients (Shao et al., 2016; Zhou Y. et al., 2020), whereas only one study including ReA patients found increased citrulline levels (Guleria et al., 2019), indicating the overall downregulation of the activity of the urea cycle in SpA. In addition, as a methyl group donor that functions in the normal metabolic cycle of methionine, betaine levels were found to be increased in two studies (Wang et al., 2016; Ahmed et al., 2019).

Creatine is an endogenous amino acid derivative synthesized from three amino acids: arginine, glycine and methionine. It is an important molecule for energy storage. Creatinine is the product of creatine during catabolism. Two studies consistently showed increased creatine levels as well as decreased creatinine levels in the serum of AS patients (Zhou Y. et al., 2020; Gupta et al., 2021). Similarly, another study detected decreased creatinine levels in the urine of AS patients (Wang et al., 2016). However, a higher level of creatinine was found in the serum of ReA patients (Guleria et al., 2019).

Despite the downregulation of tryptophan levels, the levels of its derivatives, which are involved in the kynurenine pathway, serotonin pathway and indole derivative pathway, were found to be elevated. Two independent studies observed upregulation of kynurenine levels in the serum of AS and JIA patients (Korte-Bouws et al., 2019; Eryavuz Onmaz et al., 2021). Eryavuz Onmaz et al. (2021) observed increased quinolinic acid levels and decreased levels of kynurenic acid and 3-hydroxykynurenine, which were more different in newly diagnosed AS groups than in therapy groups when compared with healthy controls. After examining colon biopsy tissues of axSpA patients, Berlinberg et al. (2021) showed elevated levels of indole-3-acetate (IAA), indole-3-acetaldehyde (I3Ald) and serotonin.

#### Alterations in the metabolism of other molecules

Aberrant choline metabolism was observed in different subtypes of SpA, but the results are inconsistent across studies. Enrolling patients with AS, Gao et al. (2008) and Wang et al. (2016) found decreased choline levels in plasma and ligament tissue, respectively. Shao et al. (2016) found the opposite results in feces. Utilizing the serum of patients, Gupta et al. (2021) found increased choline levels in SpA. However, Guleria et al. (2019) came to the opposite conclusion in ReA.

Vitamin D3 (Vit D3) and related derivatives are essential for calcium and phosphate metabolism. The downregulations of Vit D3 and derivatives were observed in three studies (Fischer et al., 2012; Comak et al., 2013; Li et al., 2019). Additionally, one study found that the levels of Vit D3 were inversely proportional to disease activity in JIA patients (Comak et al., 2013). In addition, elevated levels of phosphate in serum were detected by Gao et al. (2008) and Armstrong et al. (2014). Taken together, these results suggest an imbalance in bone metabolism in patients with SpA.

### Differences in metabolic profiling between spondyloarthritis patients and patients with other rheumatic diseases

The joint symptoms of SpA, especially PsA and ReA, can be similar to those observed in other rheumatic diseases, such as RA, OA, and gout, which have brought difficulties in clinical diagnosis and treatment. Hence, it is important to distinguish SpA from other types of arthritis by detecting specific biomarkers. Metabolomics studies conducted in SpA cases and non-SpA cases have found different metabolic profiles among different disease states, which could contribute to the identification of specific metabolites that exhibit alterations for the accurate diagnosis of SpA.

#### Spondyloarthritis cases vs. rheumatoid arthritis cases

Overall, 10 of 12 studies conducted metabolomics comparisons between SpA and rheumatoid arthritis (RA) to identify potential biomarkers to distinguish these pathologies.

Rocha et al. (2018) found that sugar was decreased in PsA synovial tissues than in RA synovial tissues by MALDI-MSI. Conversely, elevated levels of glucose in serum were observed in ReA patients (Guleria et al., 2019). Regarding lactate, the product of anaerobic glycolysis, PsA patients showed higher levels than those observed in RA patients, while two studies showed opposite results in ReA patients (Guleria et al., 2019; Souto-Carneiro et al., 2020; Dubey et al., 2021).

Lipid profiles could be used to differentiate between PsA and RA to a large extent. Higher glycerol and cholesterol levels were found in both PsA and ReA patients than in RA patients (Madsen et al., 2010; Dubey et al., 2021). Additionally, ReA patients showed lower levels of LDL and VLDL in serum and synovial fluid (Guleria et al., 2019; Dubey et al., 2021). The level of acetate, however, was highly heterogeneous among different studies. Increases or decreases in acetate levels in PsA and ReA patients were reported (Guleria et al., 2019; Souto-Carneiro et al., 2020; Bogunia-Kubik et al., 2021; Dubey et al., 2021). Levels of PUFAs were consistently found to be increased in PsA patients (Madsen et al., 2010; Souto-Carneiro et al., 2020). In addition, lysophosphatidic acids, phospholipids and sphingolipids showed higher expression levels in PsA than in RA patients (Rocha et al., 2018). Altered levels of carnitine were also observed in PsA and ReA patients, but there were gaps in relevant data that need further assessment (Toth et al., 2019; Dubey et al., 2021).

The levels of most amino acids (alanine, arginine, asparagine, aspartic acid, glutamic acid, glycine, proline, serine, tyrosine, histidine, isoleucine, leucine, lysine, methionine, threonine, tryptophan, and valine) were found to be higher in SpA patients than in RA patients, with the exception that lower levels of phenylalanine, cysteine and glutamine were observed in SpA patients (Madsen et al., 2010; Souto-Carneiro et al., 2020; Zhou Y. et al., 2020; Dubey et al., 2021). In addition, elevated levels of creatine were found in the serum of PsA patients, while the same trend for creatinine was found in ReA patients (Guleria et al., 2019; Souto-Carneiro et al., 2020).

Although there is no difference in total 25(OH)D3 values between ReA and RA patients, a lower level of phosphoric acid was reported in PsA patients, indicating more disturbed bone metabolism in SpA than RA patients (Madsen et al., 2010; Li et al., 2019).

#### Spondyloarthritis vs. other inflammatory diseases

Lower levels of glucose, lactate and acetate and higher levels of glycerol, LDL, VLDL, and choline were found in the synovial fluid of ReA patients than in OA patients. In addition, elevated levels of amino acids, including histidine, isoleucine, phenylalanine, and glutamic acid, were found in the same study group, with the exception of reduced alanine levels (Dubey et al., 2021). Another study including PsA patients also found that the synovial membrane where inflammatory infiltrates were accompanied by elevated levels of plasmalogen and phosphatidic acids but reduced levels of phosphatidylcholines (Rocha et al., 2021).

Only one study compared the different metabolic profiles observed between AS and gout patients (Jiang et al., 2013). Succinic acid and malic acid levels were lower in the serum of AS patients. Additionally, levels of amino acids, including lysine, valine, alanine, cysteine, taurine, citrulline, and creatine, were found to be reduced (Jiang et al., 2013). Both axSpA and CD-axSpA patients showed elevated levels of IAA and I3Ald than those in Crohn’s disease (CD) patients (Berlinberg et al., 2021). However, these results need further validation due to the lack of sufficient evidence.

### Dynamic alterations in the metabolic profile before and after treatment of spondyloarthritis

TNF-alpha inhibitors (TNFi) have been widely used for SpA treatment. Five of 7 enrolled studies reported dynamic alterations in metabolites after TNFi therapy in SpA (Kapoor et al., 2013; Nanus et al., 2015; Manasson et al., 2017; Butbul et al., 2020; Bogunia-Kubik et al., 2021), while the other two studies focused on the influence of conventional therapy (Funk and Becker, 2021; Gupta et al., 2021).

Kapoor et al. (2013) and Bogunia-Kubik et al. (2021) found decreased levels of isobutyrate and acetone in AS as well as decreased levels of acetate in PsA after TNFi therapy. Elevated levels of amino acids, including histidine, leucine and phenylalanine, were also found in AS patients after therapy. Another study including PsA patients receiving TNFi therapy showed lower levels of glutamine than those observed at baseline. Additionally, the two studies both found elevated creatine and creatinine levels in SpA patients after treatment. Regarding the alteration of adrenal metabolites in SpA patients receiving TNFi therapy, Butbul et al. (2020) found that the levels of most urine adrenal metabolites in JIA patients raised to normal values as in the controls. Moreover, Nanus et al. (2015) found a decreased ratio of tetrahydrocortisol (THF + 5αTHF) to tetrahydrocortisone (THE) metabolites, a decreased ratio of urinary free cortisol to urinary free cortisone, and a decreased ratio of THF to 5αTHF in the urine of PsA patients after therapy. These results suggested that TNF-α is a significant regulator of adrenal metabolism, which is further involved in the pathology of SpA.

An IL-17 inhibitor (IL-17i) is another biological agent with similar efficacy to that of TNFi used for the treatment of SpA patients, especially PsA patients. Increased levels of acetate and hexanoate were observed in PsA patients in one study (Manasson et al., 2017). Additionally, acetate and hexanoate were positively correlated with Clostridiales taxa after IL-17i therapy rather than TNFi treatment. The results indicated that IL-17i might affect the metabolism of gut microbiota (Manasson et al., 2017).

Reduced levels of glucose and elevated levels of LDL, PUFAs, isoleucine, glutamic acid and glycine were found by Gupta et al. (2021) in the serum of SpA patients after conventional therapy, which partly corrected the aberrant metabolism observed in SpA patients. In addition, in the serum of JIA patients, MTX therapy reduced the level of omega-3 unsaturated fatty acids (docosahexanoic acid and linoleic acid), which are anti-inflammatory mediators (Funk and Becker, 2021).

Taken together, these data suggest that both conventional and biological therapy of SpA can reverse the aberrant metabolic state to one that is more similar to that of the controls, albeit in an insufficient way. Nevertheless, no studies have successfully identified specific metabolites that can be used to predict treatment response.

### Differences in metabolic profiling among spondyloarthritis subtypes

Although a similar pathogenesis is shared by different subtypes of SpA, the clinical features and prognosis could be different, which could be accompanied by alterations in metabolic profiling. Gupta et al. (2021) conducted differential analysis between peripheral and axial SpA and found higher levels of lactate and N-acetyl glycoproteins as well as lower levels of LDL, PUFAs, and choline in pSpA patients than in axSpA patients. Levels of amino acids, including glutamate, proline, arginine, phenylalanine, leucine and isoleucine, were elevated in peripheral SpA, with the exception that alanine, glutamine, and histidine levels were reduced. Bogunia-Kubik et al. (2021) observed more decreased levels of creatine, lysine and acetate in the serum of PsA than in AS. These results collectively suggested distinct inflammatory states among different SpA subtypes. Inconsistently, Ahmed et al. (2019) failed to find a significant difference in the metabolic profile in both serum and synovial tissue between ReA and undifferentiated pSpA, indicating that these two subtypes could be combined into one in diagnosis with one treatment.

## Discussion

Metabolomic profiling has been increasingly applied for the comprehensive identification of the metabolic changes occurring in SpA. Here, we systematically reviewed 31 studies on the metabolomic profiling of SpA and summarized key findings on the dysregulation of major metabolic pathways (carbohydrate, lipid and amino, acid metabolism) in SpA cases compared with those observed in healthy controls and/or non-SpA cases. The dynamic alteration of metabolomic profiling of SpA cases before and after treatment, as well as differences among different subtypes, were also reviewed. More detailed altered metabolic profiles between SpA cases and non-SpA cases are summarized in Supplementary Table 3. To our knowledge, this is the first systematic review of metabolomic analysis in SpA.

### Metabolomics as a tool for precise diagnosis

By detecting different molecules involved in metabolism, metabolomics is widely applied as a tool for biomarker discovery. Here, based on the significantly altered metabolites that were identified, several studies have successfully established diagnostic models for differentiating patients with SpA from healthy participants or those with other inflammatory diseases with a high specificity and sensitivity (Madsen et al., 2010; Guleria et al., 2019; Souto-Carneiro et al., 2020; Ou et al., 2021). However, these results need to be further validated with larger study cohorts before they can be applied in the clinic. In addition, since researchers failed to identify specific biomarkers for predicting treatment response in SpA, more metabolomics analyses in longitudinal cohort studies are of great importance.

### Metabolomics as a tool for the exploration of pathogenesis

Moreover, because of the sensitivity of metabolomics, subtle alterations in biological pathways can be detected to provide insight into the mechanisms of pathogenesis that underlie SpA. After comparisons with those of healthy controls, some altered metabolic patterns are similar between SpA patients and patients with other rheumatic diseases such as RA, such as increased levels of glucose and decreased levels of lipid and membrane metabolites, suggesting that these arthritides share the same mechanisms of immunoinflammatory dysregulation, while the specific metabolites found to be altered only in SpA suggest a novel pathogenesis mechanism.

#### Elevated levels of anaerobic metabolism in spondyloarthritis patients

Elevated levels of glucose, succinic acid, malic acid, and lactate has been supported by abundant evidence, indicating the ineffective utilization of glucose and elevated anaerobic metabolism. The alteration of carbohydrate metabolism could lead to adenosine triphosphate (ATP) insufficiency. Partly caused by muscle breakdown from TNF-α-induced inflammation, an elevated level of creatine was also observed, which may play a major role in securing a continuous replenishment of the ATP pool and further lead to T-cell proliferation (Zhang et al., 2009).

#### Elevated levels of lipolysis and ectopic fat deposition in spondyloarthritis

Decreased levels of fatty acids, especially unsaturated fatty acids, suggest their augmented utilization under inflammatory conditions. Fatty acids are metabolized by β-oxidation to meet the energy demand, which is proven by the elevated level of ketone bodies. Consistently, an *in vitro* study conducted by Xu et al. (2015) found upregulations in the levels of fatty acid β-oxidation-related proteins in the fibroblast-like ligament cells of AS patients. On the other hand, the demand of proinflammatory (ω6- PUFAs) and anti-inflammatory (ω3- PUFAs) mediators is increased to perpetuate the immunometabolic response. Arachidonic acid, a representative ω6-PUFA, is correlated with disease activity in SpA (Pompeia et al., 2003; Von Hegedus et al., 2017; Coras et al., 2019b,^2021^). Conversely, ω3- PUFAs have been proposed to act as anti-inflammatory mediators and potentially affect innate and adaptive immune function, as well as the production of cytokines and reactive oxygen species (Gheita et al., 2012; Miles and Calder, 2012). The dysregulation of glycerol, triglyceride and lipoprotein can be attributed to their augmented utilization in repairing membranes of affected cells and organelles or their oxidative damage due to systemic inflammation (Castoldi et al., 2020). Interestingly, Wang et al. (2016) found that TG levels decreased in plasma but increased in ligament tissue, which may be a phenomenon associated with the deposition of ectopic fat. Since fat depositions at vertebral edges in spinal MRI were considered the most typical findings in SpA, aberrant lipid metabolism, ectopic fat deposition and further fat metaplasia may lead to new bone formation in the ligament and entheses and are thus involved in the pathogenesis of SpA (Hermann et al., 2012; Maksymowych et al., 2014). In addition, choline can promote the transport and utilization of fat; thus, the dysregulation of choline observed in several studies may be related to ectopic fat deposition and alterations in lipid metabolites (Borges Haubert et al., 2015).

#### Aberrant amino acid metabolism in spondyloarthritis patients

Aberrant amino acid metabolism is a common finding in metabolomics studies of SpA and may be associated with catabolic processes and tissue degradation for energy supplementation and protein synthesis. Proline, for example, is an important amino acid for protein synthesis, and its dysregulation may correlate with impairment of the citric acid cycle and inflammatory disease progression (Li et al., 2016).

Tryptophan is an essential amino acid and has an important role in the regulation of human immunity. Evidence has shown that in conditions characterized by immune system activation or inflammation, most circulating tryptophan is converted to kynurenine by indoleamine 2,3-dioxygenase 1 and 2 (IDO-1, IDO-2), thus activating the kynurenine pathway and leading to increased levels of proinflammatory cytokines and the dysregulation of kynurenine pathway metabolites (Kim and Jeon, 2018). Inflammatory conditions induce decreased levels of kynurenic acid, which has an antioxidant effect by performing its functions through G-protein-coupled receptor 35 (GPR35) and aryl hydrocarbon receptor (AhR) in peripheral tissues (Wang et al., 2021). Quinolinic acid performs its physiological functions by binding to N-methyl-D-aspartic acid (NMDA) receptors, which are expressed in osteoblasts and osteoclasts as well as in the central nervous system (Suva and Gaddy, 2016). Hence, increased quinolic acid levels may be associated with altered bone metabolism in patients with SpA. In addition, alterations in tryptophan metabolism in SpA may influence T cells by affecting the plasticity of effector CD4 T cells by driving them away from regulatory and toward proinflammatory CD4 T-cell phenotypes (Hatton and Weaver, 2009; Mezrich et al., 2010).

Downregulation of glutamine levels may be attributed to its consumption and conversion to glutamate by inflammatory cells, such as activated macrophages (Covarrubias et al., 2015). Glutaminolysis is considered to be one of the main sources of energy for effector T cells and facilitates Th17 development (Kono et al., 2019). Previous research reported that glutaminolysis played a key role in the cell growth of fibroblast-like synoviocytes in RA (Takahashi et al., 2017), which may also be involved in SpA.

Due to the activation of protein arginine methyltransferases (PRMTs) by inflammation and oxidative stress, the levels of methylarginine derivatives (ADMA, SDMA, and L-NMMA) increased while the levels of arginine decreased. NO, which has antiatherogenic properties and inhibits platelet aggregation and leukocyte adhesion, can be inhibited by ADMA and results in a higher risk of cardiovascular disease in SpA (Förstermann and Sessa, 2012; Zobel et al., 2017; Liu et al., 2018). Moreover, methylarginine derivatives have been shown to be related to the production of proinflammatory cytokines, such as TNF-α and IL-6 *via* the ROS/NF-κB-dependent pathway, thus contributing to the inflammatory process in turn (Schepers et al., 2011; Tripepi et al., 2011).

In addition, amino acids such as alanine, valine and threonine can activate the mammalian target of rapamycin (mTOR) pathway and induce aerobic glycolysis, innate immune activation and consequent cytokine production (Dutchak et al., 2018). T-cell activation in AS is also known to be mediated by the PI3K–AKT–mTOR pathway (Finlay et al., 2012; Perl, 2016).

#### Gut dysbiosis and altered microbial metabolites in spondyloarthritis

The microbiome, the community of microorganisms that has coevolved with human hosts, plays a pivotal role in human health and disease. Gut dysbiosis in SpA is associated with altered microbial metabolites, which can serve as messengers for microbes to communicate with each other and engage in crosstalk with host cells, contributing to the dysregulation of the host innate immune system and the pathogenesis of SpA. SCFAs are mainly produced within the intestinal lumen by bacterial fermentation of undigested dietary carbohydrates. They are the most widely studied microbiota metabolites in autoimmune diseases (Scalise et al., 2021). By increasing intestinal wall permeability and decreasing the levels of regulatory T cells and the production of Foxp3 and IL-10, decreased levels of SCFAs appear to have a crucial role in aberrant immunoregulation (Hamer et al., 2008; Smith et al., 2013). In addition, Lucas et al. (2018) found that supplementation with SCFAs in mice could increase systemic bone density, reduce bone resorption, and reduce osteoclasts, suggesting that downregulation of SCFA levels may play an important role in the aberrant bone metabolism observed in SpA.

Tryptophan metabolism occurs principally in the intestine since IDO-1 is mainly located inside intestinal cells, while tryptophanase is exclusive to bacteria. The important effects of tryptophan and its derivatives have been discussed above. Here, combined with shotgun metagenomics, Berlinberg et al. (2021) found that the microbial community in axSpA patients exhibited lower levels of tryptophan synthesis and higher levels of tryptophan metabolism toward indoles than those observed in healthy controls and patients with CD. Indoles are absorbed across the intestinal epithelium of the host and signal through either AhR or the pregnane X receptor (PXR) to modulate host responses, including barrier and immune functions (Berlinberg et al., 2021). In addition, upregulation of AhR levels has been proven to be correlated with expansion of type 3 innate lymphoid cells (ILC3s), which may link intestinal pathology to Th17 immunity in SpA (Blijdorp et al., 2019; Venken et al., 2019; Berlinberg et al., 2020).

Ethanol, which is the end-product of fermentation of different carbohydrates by gut microbiota, was found to be elevated in the feces of SpA patients. Shao et al. (2016); Vernocchi et al. (2020), and Bogunia-Kubik et al. (2021) demonstrated decreased ethanol levels after TNFi treatment, suggesting a correlation of gut dysbiosis and the perturbation of microbiota-derived metabolites with active inflammatory conditions in SpA. Similar results were also found for the contents of dehydrocholic acid and trimethylamine oxide (TMAO), which are both products of microbiota metabolism (Coras et al., 2019a; Funk and Becker, 2021).

In addition, by integrating 16S ribosomal DNA identification (16S rDNA) and metabolomics, Lorente et al. (2020) and Lv et al. (2021) found that salivary microbiota and metabolites of AS patients have more proinflammatory effects. Interestingly, the sequence of an HLA-B*27:05 ligand was observed to be highly similar to the sequence of a protein from Campylobacter, the levels of which were found to be increased in AS saliva, partly explaining the pathogenesis of HLA-B27 in SpA.

#### Other altered metabolites related to aberrant bone metabolism in spondyloarthritis

Increased activity of 11β-hydroxysteroid dehydrogenase 1 (11β-HSD1) in SpA patients after TNFi therapy was observed (Nanus et al., 2015). The results suggest that TNF-α, a regulator of glucocorticoid metabolism *in vivo*, could further contribute to the loss of bone mineral density in periarticular. Furthermore, recent study indicated that by activating 11β-HSD1, TNF-α could regulate dickkopf-1 protein (DKK-1), an inhibitor of the Wnt signaling pathway (Raza et al., 2010; Hardy et al., 2012).

In addition, disorders of bone metabolism are also reflected by the downregulation of VitD3 levels and the elevation of phosphate levels. Studies have revealed that the inflammatory condition of arthritis could influence vitamin D metabolism, which further dampens its role as a Th17-cell suppressor (Gracey et al., 2014; Harrison et al., 2020).

### Limitations

This systematic review has several limitations. First, the small cohort size in nearly half of the enrolled studies and bias between study groups due to different sex and age distributions may affect the credibility of the results. Second, the heterogeneity of results among studies may be attributed to differences in metabolite detection methods, sources of samples, and procedures of sample preprocessing and extraction. Lastly, the potential use of meta-analysis is limited since few quantitative data are available.

### Conclusion and prospects

In summary, metabolomics studies show distinct metabolic profiles between SpA and non-SpA participants, which creates a new road in finding diagnostic biomarkers for precise diagnosis and exploring pathogenesis.

Nevertheless, the diagnostic model established in several studies needs to be further validated in larger cohorts. Metabolomics studies in prospective follow-up SpA patients would also be advantageous in identifying metabolomics biomarkers for predicting the progression and treatment responses of SpA patients.

Modulating immunity and inflammation by the oral supplementation of metabolites with levels that are reduced in disease states, especially microbial-derived metabolites such as SCFAs, is considered a promising treatment for autoimmune diseases, including SpA (Rosser et al., 2020). Evidence that administration of butyrate and propionate in transgenic HLA-B27/β2m rats attenuates bowel inflammation has given future perspectives for its feasibility in SpA treatment (Asquith et al., 2017). These findings, however, need to be further confirmed by clinical trials.

Regarding the exploration of pathogenesis, integrating metabolomics with other technologies, such as transcriptomics, proteomics and metagenomics, may help to find potential key targets for treatment. In addition, more *in vitro* and *in vivo* studies are needed to determine the role of disordered metabolic molecules in the pathogenesis of SpA.

## Author contributions

TH, YP, and YBL contributed to the conception and design of the study. TH, XW, YHL, and HY contributed to data collection. TH, YP, and XW contributed to the interpretation of the data. TH and YP drafted the article. YL critically revised the manuscript. All authors approved the manuscript.
