# Corrigendum: Metabolomic analysis in spondyloarthritis: A systematic review

**DOI:** 10.3389/fmicb.2022.1100290

**Published:** 2022-12-12

**Authors:** Tianwen Huang, Yaoyu Pu, Xiangpeng Wang, Yanhong Li, Hang Yang, Yubin Luo, Yi Liu

**Affiliations:** ^1^Department of Rheumatology and Immunology, West China Hospital, Sichuan University, Chengdu, China; ^2^Rare Diseases Center, West China Hospital, Sichuan University, Chengdu, China; ^3^Institute of Immunology and Inflammation, Frontiers Science Center for Disease Related Molecular Network, West China Hospital, Chengdu, China

**Keywords:** spondyloarthritis, ankylosing spondylitis, metabolomics, biomarkers, dysbiosis

In the published article, there was an error. We mistakenly described the trend of changes in the levels of some metabolites after treatment of SpA patients.

A correction has been made to **Results**, “*Dynamic alterations in the metabolic profile before and after treatment of spondyloarthritis*,” Paragraphs 2 and 4. These sentences previously stated:

“Kapoor et al. (2013) and Bogunia-Kubik et al. (2021) found elevated levels of isobutyrate and acetone in AS as well as elevated levels of acetate in PsA after TNFi therapy. Decreased levels of amino acids, including histidine, leucine and phenylalanine, were also found in AS patients after therapy. Another study including PsA patients receiving TNFi therapy showed higher levels of glutamine than those observed at baseline. Additionally, the two studies both found decreased creatine and creatinine levels in SpA patients after treatment.”

And

“In addition, in the serum of JIA patients, MTX therapy increased the level of omega-3 unsaturated fatty acids (docosahexanoic acid and linoleic acid), which are anti-inflammatory mediators.”

The corrected sentence appears below:

“Kapoor et al. (2013) and Bogunia-Kubik et al. (2021) found decreased levels of isobutyrate and acetone in AS as well as decreased levels of acetate in PsA after TNFi therapy. Elevated levels of amino acids, including histidine, leucine and phenylalanine, were also found in AS patients after therapy. Another study including PsA patients receiving TNFi therapy showed lower levels of glutamine than those observed at baseline. Additionally, the two studies both found elevated creatine and creatinine levels in SpA patients after treatment.”

And

“In addition, in the serum of JIA patients, MTX therapy reduced the level of omega-3 unsaturated fatty acids (docosahexanoic acid and linoleic acid), which are anti-inflammatory mediators.”

The authors apologize for this error and state that this does not change the scientific conclusions of the article in any way. The original article has been updated.
