# Supplementary material for: Metabolomic analysis in spondyloarthritis: A systematic review
Source: Front Microbiol. 2022 Sep 2;13:965709. doi: 10.3389/fmicb.2022.965709 (PMC9479008; doi:10.3389/fmicb.2022.965709)
Supplement: Supplementary file 1 [file Table_1.DOCX]

**Search strategy**

**Pubmed：**

("spondylarthritis"[MeSH Terms] OR "spondylarthritis"[All Fields] OR "spondyloarthritis"[All Fields] OR ("spondylitis, ankylosing"[MeSH Terms] OR ("spondylitis"[All Fields] AND "ankylosing"[All Fields]) OR "ankylosing spondylitis"[All Fields] OR ("ankylosing"[All Fields] AND "spondylitis"[All Fields])) OR ("arthritis, reactive"[MeSH Terms] OR ("arthritis"[All Fields] AND "reactive"[All Fields]) OR "reactive arthritis"[All Fields] OR ("reactive"[All Fields] AND "arthritis"[All Fields])) OR ("arthritis, reactive"[MeSH Terms] OR ("arthritis"[All Fields] AND "reactive"[All Fields]) OR "reactive arthritis"[All Fields] OR ("reiter’s"[All Fields] AND "syndrome"[All Fields]) OR "reiter’s syndrome"[All Fields]) OR ("arthritis, psoriatic"[MeSH Terms] OR ("arthritis"[All Fields] AND "psoriatic"[All Fields]) OR "psoriatic arthritis"[All Fields] OR ("psoriatic"[All Fields] AND "arthritis"[All Fields])) OR ("arthritis, juvenile"[MeSH Terms] OR ("arthritis"[All Fields] AND "juvenile"[All Fields]) OR "juvenile arthritis"[All Fields] OR ("juvenile"[All Fields] AND "idiopathic"[All Fields] AND "arthritis"[All Fields]) OR "juvenile idiopathic arthritis"[All Fields]) OR (("inflammatory bowel diseases"[MeSH Terms] OR ("inflammatory"[All Fields] AND "bowel"[All Fields] AND "diseases"[All Fields]) OR "inflammatory bowel diseases"[All Fields] OR ("inflammatory"[All Fields] AND "bowel"[All Fields] AND "disease"[All Fields]) OR "inflammatory bowel disease"[All Fields]) AND ("arthritis"[MeSH Terms] OR "arthritis"[All Fields] OR "arthritides"[All Fields] OR "polyarthritides"[All Fields])) OR (("undifferentiate"[All Fields] OR "undifferentiated"[All Fields] OR "undifferentiating"[All Fields] OR "undifferentiation"[All Fields]) AND ("spondylarthritis"[MeSH Terms] OR "spondylarthritis"[All Fields] OR "spondyloarthritis"[All Fields]))) AND ("metabolome"[MeSH Terms] OR "metabolome"[All Fields] OR "metabolomes"[All Fields] OR "metabolomics"[MeSH Terms] OR "metabolomics"[All Fields] OR "metabolomic"[All Fields])

**Web of Science:**

(TS=(spondyloarthritis) OR TS=(ankylosing spondylitis) OR TS=(reactive arthritis) OR TS=(Reiter's syndrome) OR TS=(psoriatic arthritis) OR TS=(juvenile idiopathic arthritis) OR (TS=(inflammatory bowel disease) AND TS=(arthritis)) OR TS=(undifferentiated spondyloarthritis)) AND TS=(metabolomic)

**Medline:**

1. exp Spondylarthropathies/

2. (Spondylarthr* or Spondyloarthr* or Spondyloarthritis).tw,ab,kw.

3. 1 or 2

4. exp Spondylitis, Ankylosing/

5. (Bechtere* or (ankylo* and spondyl*) or (Marie and struempell*)).tw,ab,kw.

6. 4 or 5

7. exp Arthritis, Psoriatic/

8. (psoria* and (arthriti* or arthropath*)).tw,ab,kw.

9. 7 or 8

10. exp Arthritis, Infectious/

11. (reiter* and (disease or syndrome)).tw,ab,kw.

12. ((sexual* or chlamydia or yersinia or postyersinia or postdysenteric or salmonella or Shigella or b27 or postinfectious or post infectious) and arthriti*).tw,ab,kw

13. exp Arthritis, Reactive/

14. 10 or 11 or 12 or 13

15. exp Arthritis, Juvenile/

16. Enthesitis Related Arthritis.tw,ab,kw.

17. exp Inflammatory Bowel Diseases/

18. Inflammatory Bowel Diseases.tw,ab,kw.

19. 17 or 18

20. exp Arthritis/

21. 19 and 20

22. undifferentiated spondyloarthritis.tw,ab,kw.

23. 3 or 6 or 9 or 14 or 15 or 16 or 21 or 22

24. exp Metabolomics/

25. metabo*.tw,ab,kw.

26. biochemical marker*.tw,ab,kw.

27. glucose metabolism.tw,ab,kw.

28. amino acid metabolism.tw,ab,kw.

29. exp Carbohydrate Metabolism/

30. carbohydrate metabolism.tw,ab,kw.

31. 29 or 30

32. chemometric*.tw,ab,kw.

33. exp Lipidomics/

34. lipidomic*.tw,ab,kw.

35. 33 or 34

36. 24 or 25 or 26 or 27 or 28 or 31 or 32 or 35

37. multiomic*.tw,ab,kw.

38. profiling.tw,ab,kw.

39. exp Mass Spectrometry/

40. exp Magnetic Resonance Spectroscopy/

41. exp Spectrum Analysis/

42. exp Spectrophotometry/

43. (LC-MS or GCMS).tw,ab,kw.

44. (NMR or HPLC).tw,ab,kw.

45. 37 or 38 or 39 or 40 or 41 or 42 or 43 or 44

46. 23 and 36 and 45

**Embase:**

1. exp spondyloarthropathy/

2. (Spondylarthr* or Spondyloarthr* or Spondyloarthritis).tw,ab,kw.

3. 1 or 2

4. exp ankylosing spondylitis/

5. (Bechtere* or (ankylo* and spondyl*) or (Marie and struempell*)).tw,ab,kw.

6. 4 or 5

7. exp psoriatic arthritis/

8. (psoria* and (arthriti* or arthropath*)).tw,ab,kw.

9. 7 or 8

10. exp infectious arthritis/

11. (reiter* and (disease or syndrome)).tw,ab,kw.

12. ((sexual* or chlamydia or yersinia or postyersinia or postdysenteric or salmonella or Shigella or b27 or postinfectious or post infectious) and arthriti*).tw,ab,kw.

13. exp reactive arthritis/

14. 10 or 11 or 12 or 13

15. exp juvenile rheumatoid arthritis/

16. Enthesitis Related Arthritis.tw,ab,kw.

17. exp inflammatory bowel disease/

18. Inflammatory Bowel Diseases.tw,ab,kw.

19. 17 or 18

20. exp arthritis/

21. 19 and 20

22. undifferentiated spondyloarthritis.tw,ab,kw.

23. 3 or 6 or 9 or 14 or 15 or 16 or 21 or 22

24. exp metabolomics/

25. metabo*.tw,ab,kw.

26. biochemical marker*.tw,ab,kw.

27. exp glucose metabolism/

28. glucose metabolism.tw,ab,kw.

29. 27 or 28

30. exp amino acid metabolism/

31. amino acid metabolism.tw,ab,kw.

32. 30 or 31

33. exp carbohydrate metabolism/

34. carbohydrate metabolism.tw,ab,kw.

35. 33 or 34

36. exp chemometric analysis/

37. chemometric*.tw,ab,kw.

38. 36 or 37

39. exp lipidomics/

40. lipidomic*.tw,ab,kw.

41. 39 or 40

42. 24 or 25 or 26 or 29 or 32 or 35 or 38 or 41

43. multiomic*.tw,ab,kw.

44. profiling.tw,ab,kw.

45. exp mass spectrometry/

46. exp nuclear magnetic resonance spectroscopy/

47. exp spectroscopy/

48. exp spectrophotometry/

49. (LC-MS or GCMS).tw,ab,kw.

50. (NMR or HPLC).tw,ab,kw.

51. 43 or 44 or 45 or 46 or 47 or 48 or 49 or 50

52. 23 and 42 and 51
