# Supplementary material for: Metabolomic analysis in spondyloarthritis: A systematic review
Source: Front Microbiol. 2022 Sep 2;13:965709. doi: 10.3389/fmicb.2022.965709 (PMC9479008; doi:10.3389/fmicb.2022.965709)
Supplement: Supplementary file 2 [file Table_2.DOCX]

**Quality assessment of Included Study by the Newcastle Ottawa Scale**

| **Study** | **Selection** | | | | **Comparability for important factors** | **Exposure** | | | **Scores** |
| --- | --- | --- | --- | --- | --- | --- | --- | --- | --- |
|  | Adequate definition of cases | Representativeness of cases | Selection of controls | Definition of controls |  | Ascertainment of exposure | Same method of ascertainment for cases and controls | Non-response rate |  |
| Gao *et al.* 2008 | NR | ☆ | ☆ | ☆ | ☆☆ | NR | NR | ☆ | 6 |
| Madsen *et al.* 2010 | ☆ | ☆ | NR | ☆ | ☆ | NR | NR | ☆ | 5 |
| Fischer *et al*. 2012 | ☆ | ☆ | NR | ☆ | ☆☆ | NR | NR | ☆ | 6 |
| Jiang *et al.* 2013 | ☆ | ☆ | ☆ | ☆ | ☆ | NR | NR | ☆ | 6 |
| Kapoor *et al.* 2013 | ☆ | ☆ | NR | NR | ☆☆ | NR | NR | ☆ | 5 |
| Armstrong *et al.* 2014 | ☆ | ☆ | NR | ☆ | ☆☆ | NR | NR | ☆ | 6 |
| Zeft *et al.* 2014 | ☆ | ☆ | NR | ☆ | NR | NR | NR | ☆ | 4 |
| Chen *et al.* 2015 | ☆ | ☆ | ☆ | ☆ | ☆☆ | NR | NR | ☆ | 7 |
| Nanus *et al.* 2015 | ☆ | ☆ | NR | NR | ☆☆ | NR | NR | ☆ | 5 |
| Shao *et al.* 2016 | NR | ☆ | ☆ | ☆ | ☆☆ | NR | NR | ☆ | 6 |
| Stoll *et al.* 2016 | ☆ | ☆ | ☆ | ☆ | ☆ | NR | NR | ☆ | 6 |
| Wang *et al.* 2016 | ☆ | ☆ | ☆ | ☆ | ☆☆ | NR | NR | ☆ | 7 |
| Ahmed *et al.* 2019 | ☆ | ☆ | NR | ☆ | ☆ | NR | NR | ☆ | 5 |
| Guleria *et al.* 2019 | ☆ | ☆ | NR | ☆ | ☆☆ | NR | NR | ☆ | 6 |
| He *et al.* 2019 | ☆ | ☆ | NR | ☆ | ☆☆ | NR | NR | ☆ | 6 |
| Li *et al.* 2019 | NR | ☆ | NR | ☆ | NR | NR | NR | ☆ | 3 |
| Butbul *et al.* 2020 | NR | ☆ | NR | ☆ | ☆☆ | NR | NR | ☆ | 5 |
| Muhammed *et al.* 2020 | ☆ | ☆ | NR | ☆ | NR | NR | NR | ☆ | 4 |
| Souto-Carneiro *et al.* 2020 | ☆ | ☆ | NR | ☆ | NR | NR | NR | ☆ | 4 |
| Vernocchi *et al.* 2020 | ☆ | ☆ | NR | ☆ | ☆ | NR | NR | ☆ | 5 |
| Zhou *et al.* 2020 | ☆ | ☆ | ☆ | ☆ | ☆ | NR | NR | ☆ | 6 |
| Berlinberg *et al.* 2021 | ☆ | ☆ | ☆ | ☆ | ☆☆ | NR | NR | ☆ | 7 |
| Bogunia-Kubik *et al.* 2021 | ☆ | ☆ | NR | ☆ | ☆ | NR | NR | ☆ | 5 |
| Dubey *et al.* 2021 | ☆ | ☆ | NR | NR | NR | NR | NR | ☆ | 3 |
| Eryavuz Onmaz *et al.* 2021 | ☆ | ☆ | ☆ | ☆ | ☆☆ | NR | NR | ☆ | 7 |
| Funk *et al.* 2021 | ☆ | ☆ | NR | NR | ☆☆ | NR | NR | ☆ | 5 |
| Gupta *et al.* 2021 | ☆ | ☆ | ☆ | ☆ | ☆☆ | NR | NR | ☆ | 7 |
| Lv *et al.* 2021 | ☆ | ☆ | NR | ☆ | ☆☆ | NR | NR | ☆ | 6 |
| Onmaz et al. 2021 | ☆ | ☆ | NR | ☆ | ☆☆ | NR | NR | ☆ | 6 |
| Ou *et al.* 2021 | ☆ | ☆ | ☆ | ☆ | ☆☆ | NR | NR | ☆ | 7 |
| Rocha *et al.* 2021 | ☆ | ☆ | NR | ☆ | NR | NR | NR | ☆ | 4 |

NR, not reported
