# Supplementary material for: Metabolomic analysis in spondyloarthritis: A systematic review
Source: Front Microbiol. 2022 Sep 2;13:965709. doi: 10.3389/fmicb.2022.965709 (PMC9479008; doi:10.3389/fmicb.2022.965709)
Supplement: Supplementary file 3 [file Table_3.DOCX]

**Summary of altered metabolic profiles between SpA cases and non-SpA cases**

- **Carbohydrate metabolism**

**SpA VS HC**

| Study | Sample type | Analytical technique | Subtype | Glucose | Glyceraldehyde | Pyruvate | Lactate | Succinic acid | Fumaric acid | Malic acid | Others |
| --- | --- | --- | --- | --- | --- | --- | --- | --- | --- | --- | --- |
| Gupta et al. 2021 | Serum | ^1^ H NMR | SpA |  |  |  | ↑ |  |  |  |  |
| Gao et al. 2008 | Plasma | LC-MS & GC-MS | AS | ↑ |  |  |  |  |  |  |  |
| Jiang et al. 2013 | Serum | GC-TOF MS & UPLC-QTOF MS | AS |  | ↑ |  |  |  | ↑ dihydroxyfumaric acid |  |  |
| Shao et al. 2016 | Feces | ^1^ H NMR | AS | ↑ |  |  |  |  | ↑ |  | ↓ UDP-glucose |
| He et al. 2019 | Feces | GC-MS | AS |  |  |  |  |  |  |  | ↑  AS VS HC:  gluconic acid  AS males VS females:  propanedioic acid |
| Lv et al. 2021 | Saliva | GC-MS | AS |  |  |  |  |  | ↑ | ↑ |  |
| Onmaz et al. 2021 | Serum | LC-MS | AS | ↑ |  |  |  |  |  |  |  |
| Armstrong et al. 2014 | Serum | GC-TOF MS | PsA |  |  |  |  |  |  |  | ↑ glucuronic acid |
| Guleria et al. 2019 | Serum | ^1^ H NMR | ReA | ↑ |  | ↓ |  |  |  |  |  |
| Ahmed et al. 2019 | Serum | ^1^ H NMR | ReA  uSpA |  |  | ↓ |  |  |  |  | ↑ mannose  ↓ malonate |

**SpA VS Other rheumatic diseases**

| Study | Sample type | Analytical technique | Subtype | Glucose | Glyceraldehyde | Pyruvate | Lactate | Succinic acid | Fumaric acid | Malic acid | Others |
| --- | --- | --- | --- | --- | --- | --- | --- | --- | --- | --- | --- |
| Madsen et al. 2010 | Plasma | LC-MS & GC-MS | PsA VS RA |  |  |  |  | ↓ |  |  |  |
| Rocha et al. 2017/2018 | Synovial membrane | MALDI-MSI | PsA VS RA | ↓ sugars including N-acetylhexosamine 6-sulfate, glucuronic acid 1-phosphate and N-acetylneuraminic acid | | | | | | | |
| Souto-Carneiro et al. 2020 | Serum | ^1^ H NMR | PsA VS negRA |  |  |  | ↑ |  |  |  |  |
| Guleria et al. 2019 | Serum | ^1^ H NMR | ReA VS RA | ↑ |  | ↑ | ↑ |  |  |  |  |
| Dubey et al. 2021 | Synovial fluid | ^1^ H NMR | ReA VS RA |  |  |  | ↓ |  |  |  |  |
| Dubey et al. 2021 | Synovial fluid | ^1^ H NMR | ReA VS OA | ↓ |  |  | ↓ |  |  |  |  |
| Jiang et al. 2013 | Serum | GC-TOF MS & UPLC-QTOF MS | AS VS GA |  |  |  |  | ↓ |  | ↓ | ↓ sucrose |

GA, gout arthritis

**Metabolic profile of SpA before treatment, compared with SpA after treatment**

| Study | Sample type | Analytical technique | Subtype | Glucose | Glyceraldehyde | Pyruvate | Lactate | Succinic acid | Fumaric acid | Malic acid | others |
| --- | --- | --- | --- | --- | --- | --- | --- | --- | --- | --- | --- |
| Gupta et al. 2021 | Serum | ^1^ H NMR | SpA | ↑ |  |  |  |  |  |  |  |

- **Lipid metabolism**

**SpA VS HC**

| Study | Sample type | Analytical technique | Subtype | Glycerol | Triglycerides | Cholesterol | Lipoproteins | Saturated fatty acid | Unsaturated fattyacid | Phospholipid | Ketone  bodies |
| --- | --- | --- | --- | --- | --- | --- | --- | --- | --- | --- | --- |
| Berlinberg et al. 2021 | Colon biopsies | LC-MS | axSpA |  |  |  |  |  | ↓ omega 3 compounds：docosahexaenoic acid; eicosapentaenoic acid |  |  |
| Gupta et al. 2021 | Serum | ^1^ H NMR | SpA |  |  |  | ↓ LDL/VLDL | ↑ acetate | ↓ PUFA |  | ↓ acetone |
| Gao et al. 2008 | Plasma | LC-MS & GC-MS | AS | ↑ |  |  |  |  |  |  |  |
| Chen et al. 2015 | Serum | GC-MS | AS |  |  |  |  |  | Serum free fatty acid profiles:  ↑ arachidonic acid  Serum esterified fatty acid profiles:  ↓ arachidonic acid |  |  |
| Shao et al. 2016 | Feces | ^1^ H NMR | AS |  |  |  |  | ↓ butyrate, propionate  ↑ formate |  |  |  |
| Wang et al. 2016 | Plasma, Urine, ligament tissue | ^1^ H NMR | AS | ↑ plasma | ↑ ligament tissue  ↓ plasma |  |  | ↓ urine: butyrate |  |  | ↑ plasma: 3-hydroxybutyrate, acetoacetate, acetone |
| He et al. 2019 | Feces | GC-MS | AS |  |  | ↓ AS male VS HC male |  | ↓ acetate  AS female patients VS HC female |  |  |  |
| Lv et al. 2021 | Saliva | GC-MS | AS |  |  |  |  |  |  | ↓ PE | ↑ 3-hydroxybutyric acid |
| Onmaz et al. 2021 | Serum | LC-MS | AS |  | ↑ | ↑ | ↑ LDL  ↓ HDL |  |  |  |  |
| Guleria et al. 2019 | Serum | ^1^ H NMR | ReA |  |  |  | ↓ LDL, VLDL | ↑ acetate | ↓ PUFA |  | ↑ acetoacetate  ↓ acetone |
| Ahmed et al. 2019 | Serum | ^1^ H NMR | ReA  uSpA |  |  |  |  |  |  |  | ↑ 3-hydroxybutyrate, acetone |

PUFA, polyunsaturated fatty acid; PE, Phosphoethanolamine

**SpA VS Other rheumatic diseases**

| Study | Sample type | Analytical technique | Subtype | Glycerol | Triglycerides | Cholesterol | Lipoproteins | Saturated fatty acid | Unsaturated fattyacid | Phospholipid | Ketone bodies |
| --- | --- | --- | --- | --- | --- | --- | --- | --- | --- | --- | --- |
| Madsen et al. 2010 | Plasma | LC-MS & GC-MS | PsA VS RA | ↑ |  | ↑ |  | ↓ heptanoic acid | ↑ arachidonic acid |  |  |
| Souto-Carneiro et al. 2020 | Serum | ^1^ H NMR | PsA VS negRA |  |  |  |  | ↑ acetate, L3/L1, L5/L1 | ↑ L6/L1 |  |  |
| Bogunia-Kubik et al. 2021 | Serum | ^1^ H NMR | PsA VS RA |  |  |  |  | ↓ acetate |  |  |  |
| Rocha et al. 2017/2018 | Synovial membrane | MALDI-MSI | PsA VS RA |  |  |  |  | ↑ |  | ↑ LPA and sphingolipids |  |
| Dubey et al. 2021 | Synovial fluid | ^1^ H NMR | ReA VS RA | ↑ |  |  | ↓ LDL | ↓ acetate |  |  | ↑ acetone |
| Guleria et al. 2019 | Serum | ^1^ H NMR | ReA VS RA |  |  |  | ↓ LDL, VLDL | ↑ acetate |  |  | ↑ acetoacetate  ↓ acetone |
| Dubey et al. 2021 | Synovial fluid | ^1^ H NMR | ReA VS OA | ↑ |  |  | ↑  LDL, VLDL | ↓ acetate |  |  |  |
| Rocha et al. 2021 | Synovial membrane | MALDI-MSI | PsA VS OA |  |  |  |  |  |  | ↑ PE-based plasmalogens, PA  ↓ PC |  |

LPA, lysophosphatidic acid; PE, Phosphoethanolamine; PA, phosphatidic acids; PC, phosphatidylcholines

**Metabolic profile of SpA before treatment, compared with SpA after treatment**

| Study | Sample type | Analytical technique | Subtype | Glycerol | Triglycerides | Cholesterol | Lipoproteins | Saturated fatty acid | Unsaturated fattyacid | phospholipid | Ketone bodies |
| --- | --- | --- | --- | --- | --- | --- | --- | --- | --- | --- | --- |
| Gupta et al. 2021 | Serum | ^1^ H NMR | SpA |  |  |  | ↓ LDL/VLDL |  | ↓ PUFA |  |  |
| Bogunia-Kubik et al. 2021 | Serum | ^1^ H NMR | AS  PsA |  |  |  |  | AS  ↑ isobutyrate  PsA  ↑ acetate |  |  | ↑ acetone |
| Funk et al. 2021 | Plasma | GC-TOF MS;  Q-TOF-MS | JIA |  |  |  |  |  | ↑ docosahexanoic acid and linoleic acid |  |  |

PUFA: polyunsaturated fatty acid.

- **Amino acid metabolism**

**SpA VS HC**

| Study | Sample type | Analytical technique | Subtype | Essential amino acid | | | | | | | | | Non-essential amino acid | | | | | | | | | | |
| --- | --- | --- | --- | --- | --- | --- | --- | --- | --- | --- | --- | --- | --- | --- | --- | --- | --- | --- | --- | --- | --- | --- | --- |
|  |  |  |  | His | Ile | Leu | Lys | Met | Phe | Thr | Trp | Val | Ala | Arg | Asn | Asp | Cys | Gln | Glu | Gly | Pro | Ser | Tyr |
| Gao et al. 2008 | Plasma | LC-MS & GC-MS | AS |  |  |  |  |  | ↑ |  | ↓ |  |  |  |  |  | ↑ |  |  |  | ↑ |  |  |
| Jiang et al. 2013 | Serum | GC-TOF MS & UPLC-QTOF MS | AS |  |  |  |  |  |  |  |  |  |  |  |  | ↑ |  |  |  |  |  | ↑ |  |
| Wang et al. 2016 | Plasma  Urine | ^1^ H NMR | AS |  |  | ↓  plasma |  | ↑  plasma |  |  |  | ↓  plasma | ↓  plasma |  |  |  |  |  | ↓  plasma  urine | ↑  urine |  |  |  |
| Zhou et al. 2020 | Serum | UPLC-TQ-MS | AS | ↑ | ↑ | ↑ | ↑ | ↑ | ↓ |  | ↑ | ↑ | ↑ |  | ↓ |  |  | ↓ | ↓ | ↓ | ↓ | ↓ | ↑ |
| Eryavuz Onmaz et al. 2021 | Serum | LC-MS | AS |  |  |  |  |  |  |  | ↓ |  |  |  |  |  |  |  |  |  |  |  |  |
| Onmaz et al. 2021 | Serum | LC-MS | AS |  |  |  |  |  |  |  |  |  |  | ↓ |  |  |  |  |  |  |  |  |  |
| Shao et al. 2016 | Feces | ^1^ H NMR | AS |  |  | ↓ |  | ↓ | ↓ | ↑ | ↑ | ↓ | ↑ |  |  |  |  |  |  |  | ↑ |  |  |
| He et al. 2019 | Feces | GC-MS | AS |  |  |  |  |  |  |  |  |  |  |  |  |  |  |  |  |  |  | ↑ |  |
| Lv et al. 2021 | Saliva | GC-MS | AS |  |  |  | ↑ |  |  | ↑ |  | ↑ | ↑ |  |  |  |  |  | ↑ | ↑ | ↑ | ↑ | ↑ |
| Stoll et al. 2016 | Feces | LC-MS | JIA/ERA |  |  |  |  |  |  |  | ↓ |  |  |  |  |  |  |  |  |  |  |  |  |
| Guleria et al. 2019 | Serum | ^1^ H NMR | ReA | ↑ | ↑ | ↑ | ↑ | ↑ | ↑ |  |  | ↑ |  |  |  |  |  | ↑ | ↑ | ↑ |  |  |  |
| Ahmed et al. 2019 | Serum | ^1^ H NMR | ReA  uSpA |  |  |  |  |  | ↑ |  |  |  |  |  |  | ↑ |  |  |  |  |  |  |  |
| Gupta et al. 2021 | Serum | ^1^ H NMR | SpA | ↑ | ↑ | ↑ | ↑ | ↓ | ↑ |  |  | ↑ | ↑ | ↑ |  |  |  | ↑ | ↑ |  | ↑ |  |  |

His, Histidine; Ile, Isoleucine; Leu, Leucine; Lys, Lysine; Met, Methionine; Phe, Phenylalanine; Thr, Threonine; Trp, Tryptophan; Val, Valine; Ala, Alanine; Arg, Arginine; Asn, Asparagine; Asp, Asparticacid; Cys, Cysteine; Gln, Glutamine; Glu, Glutamicacid; Gly, Glycine; Pro, Proline; Ser, Serine; Tyr, Tyrosine.

**SpA VS Other rheumatic diseases**

| Study | Sample type | Analytical technique | Subtype | Essential amino acid | | | | | | | | | Non-essential amino acid | | | | | | | | | | |
| --- | --- | --- | --- | --- | --- | --- | --- | --- | --- | --- | --- | --- | --- | --- | --- | --- | --- | --- | --- | --- | --- | --- | --- |
|  |  |  |  | His | Ile | Leu | Lys | Met | Phe | Thr | Trp | Val | Ala | Arg | Asn | Asp | Cys | Gln | Glu | Gly | Pro | Ser | Tyr |
| Zhou et al. 2020 | Serum | UPLC-TQ-MS | AS VS RA | ↑ | ↑ | ↑ | ↑ | ↑ | ↓ | ↑ | ↑ | ↑ | ↑ | ↑ | ↑ |  |  |  |  | ↑ |  | ↑ |  |
| Madsen et al. 2010 | Plasma | LC-MS & GC-MS | PsA VS RA | ↑ |  |  |  |  |  |  |  |  |  |  |  | ↑ | ↓ | ↓ | ↑ |  |  | ↑ |  |
| Souto-Carneiro et al. 2020 | Serum | ^1^ H NMR | PsA VS negRA |  |  | ↑ |  |  | ↓ | ↑ |  | ↑ | ↑ |  |  |  |  |  |  |  |  |  |  |
| Bogunia-Kubik et al. 2021 | Serum | ^1^ H NMR | PsA VS AS&RA |  |  |  | ↓ |  |  |  |  |  |  |  |  |  |  |  |  |  |  |  |  |
| Guleria et al. 2019 | Serum | ^1^ H NMR | ReA VS RA | ↑ |  | ↑ | ↑ | ↑ | ↑ |  |  | ↑ |  |  |  |  |  | ↑ | ↑ |  |  |  | ↑ |
| Muhammed et al. 2020 | Synovial fluid | ^1^ H NMR | ReA/uSpA VS RA&OA |  |  |  |  |  | ↑ |  |  |  |  |  |  |  |  |  |  |  |  |  |  |
| Dubey et al. 2021 | Synovial fluid | ^1^ H NMR | ReA VS RA |  |  |  |  |  |  |  |  |  | ↓ |  |  |  |  | ↓ |  | ↓ |  |  |  |
| Dubey et al. 2021 | Synovial fluid | ^1^ H NMR | ReA VS OA | ↑ | ↑ |  |  |  |  |  |  |  | ↓ |  |  |  |  |  | ↑ |  |  |  |  |
| Jiang et al. 2013 | Serum | GC-TOF MS & UPLC-QTOF MS | AS VS GA |  |  |  | ↓ |  |  |  |  | ↓ | ↓ |  |  |  | ↓ |  |  |  |  |  |  |

His, Histidine; Ile, Isoleucine; Leu, Leucine; Lys, Lysine; Met, Methionine; Phe, Phenylalanine; Thr, Threonine; Trp, Tryptophan; Val, Valine; Ala, Alanine; Arg, Arginine; Asn, Asparagine; Asp, Asparticacid; Cys, Cysteine; Gln, Glutamine; Glu, Glutamicacid; Gly, Glycine; Pro, Proline; Ser, Serine; Tyr, Tyrosine.

**Metabolic profile of SpA before treatment, compared with SpA after treatment**

| Study | Sample type | Analytical technique | Subtype | Essential amino acid | | | | | | | | | Non-essential amino acid | | | | | | | | | | |
| --- | --- | --- | --- | --- | --- | --- | --- | --- | --- | --- | --- | --- | --- | --- | --- | --- | --- | --- | --- | --- | --- | --- | --- |
|  |  |  |  | His | Ile | Leu | Lys | Met | Phe | Thr | Trp | Val | Ala | Arg | Asn | Asp | Cys | Gln | Glu | Gly | Pro | Ser | Tyr |
| Gupta et al. 2021 | Serum | ^1^ H NMR | SpA |  | ↑ |  |  |  |  |  |  |  |  |  |  |  |  |  | ↑ | ↑ |  |  |  |
| Bogunia-Kubik et al. 2021 | Serum | ^1^ H NMR | AS | ↓ |  | ↓ |  |  | ↓ |  |  |  |  |  |  |  |  |  |  |  |  |  |  |
| Kapoor et al. 2013 | Urine | ^1^ H NMR | PsA |  |  |  |  |  |  |  |  |  |  |  |  |  |  | ↑ |  |  |  |  |  |

His, Histidine; Ile, Isoleucine; Leu, Leucine; Lys, Lysine; Met, Methionine; Phe, Phenylalanine; Thr, Threonine; Trp, Tryptophan; Val, Valine; Ala, Alanine; Arg, Arginine; Asn, Asparagine; Asp, Asparticacid; Cys, Cysteine; Gln, Glutamine; Glu, Glutamicacid; Gly, Glycine; Pro, Proline; Ser, Serine; Tyr, Tyrosine.

- **Other altered metabolites**

**Choline metabolism**

**SpA VS HC**

| Study | Sample type | Analytical technique | Subtype | Metabolites |
| --- | --- | --- | --- | --- |
| Gupta et al. 2021 | Serum | ^1^ H NMR | SpA | ↑ choline |
| Gao et al. 2008 | Plasma | LC-MS & GC-MS | AS | ↓ phosphocholines |
| Shao et al. 2016 | Feces | ^1^ H NMR | AS | ↑ choline |
| Wang et al. 2016 | ligament tissue | ^1^ H NMR | AS | ↓ choline |
| Guleria et al. 2019 | Serum | ^1^ H NMR | ReA | ↓ choline |

**SpA VS Other rheumatic diseases**

| Study | Sample type | Analytical technique | Subtype | Metabolites |
| --- | --- | --- | --- | --- |
| Souto-Carneiro et al. 2020 | Serum | ^1^ H NMR | PsA VS negRA | ↑choline |
| Guleria et al. 2019 | Serum | ^1^ H NMR | ReA VS RA | ↓choline |
| Dubey et al. 2021 | Synovial fluid | ^1^ H NMR | ReA VS RA/OA | ↓choline |

**VitD3 metabolism**

**SpA VS HC**

| Study | Sample type | Analytical technique | Subtype | Metabolites |
| --- | --- | --- | --- | --- |
| Fischer et al. 2012 | Serum | LC-MS | AS | ↓ (23S,25R)-25-hydroxyvitamin D3 |
| Li et al. 2019 | Serum,  synovial fluid | LC-MS | ReA | ↓ serum: 3-epi-25(OH)D3 25(OH)D2  ↓synovial fluid: 3-epi-25(OH)D3 |

**Carnitine metabolism**

**SpA VS HC**

| Study | Sample type | Analytical technique | Subtype | Metabolites |
| --- | --- | --- | --- | --- |
| Zhou et al. 2020 | Serum | UPLC-TQ-MS | AS | ↓acetylcarnitine |
| Jiang et al. 2013 | Serum | GC-TOF MS & UPLC-QTOF MS | AS | ↓4,8-dimethylnonanoyl carnitine |

**SpA VS Other rheumatic diseases**

| study | Sample type | Analytical technique | Subtype | Metabolites |
| --- | --- | --- | --- | --- |
| Dubey et al. 2021 | Synovial fluid | ^1^ H NMR | ReA | ↓carnitine |

**Creatine metabolism**

**SpA VS HC**

| Study | Sample type | Analytical technique | Subtype | Metabolites |
| --- | --- | --- | --- | --- |
| Zhou et al. 2020 | Serum | UPLC-TQ-MS | AS | ↑creatine  ↓ creatinine |
| Gupta et al. 2021 | Serum | ^1^ H NMR | AS | ↑ creatine/creatinine  ↓ creatinine |
| Wang et al. 2016 | Urine | ^1^ H NMR | AS | ↓urine creatinine |
| Guleria et al. 2019 | Serum | ^1^ H NMR | ReA | ↑creatinine |

**SpA VS Other rheumatic diseases**

| Study | Sample type | Analytical technique | Subtype | Metabolites |
| --- | --- | --- | --- | --- |
| Souto-Carneiro et al. 2020 | Serum | ^1^ H NMR | PsA VS negRA | ↑ creatine |
| Bogunia-Kubik et al. 2021 | Serum | ^1^ H NMR | PSA VS AS& RA | ↓creatine |
| Guleria et al. 2019 | Serum | ^1^ H NMR | ReA VS RA | ↑creatinine |
| Jiang et al. 2013 | Serum | GC-TOF MS & UPLC-QTOF MS | AS VS GA | ↓creatine |

GA, gout arthritis.
